# A Nomogram for Predicting Portal Hypertensive Gastropathy in Patients With Liver Cirrhosis: A Retrospective Analysis

**DOI:** 10.3389/fmed.2022.834159

**Published:** 2022-02-18

**Authors:** WenSheng Wang, ZhiYong Mu, GuangXi Zhu, Tao Wang, ShuJie Lai, Yan Guo, XinRu Yin, LiangZhi Wen, DongFeng Chen

**Affiliations:** Department of Gastroenterology, Daping Hospital, Army Medical University, Chongqing, China

**Keywords:** cirrhosis, portal hypertensive gastropathy, logistic regression, nomogram, predictors

## Abstract

**Background:**

There is an urgent need for non-invasive methods for predicting portal hypertensive gastropathy (PHG). This study aims to develop and validate a non-invasive method based on clinical parameters for predicting PHG in patients with liver cirrhosis (LC).

**Methods:**

The overall survival (OS) and hepatocellular carcinoma (HCC)-free survival were evaluated in LC patients, both with and without PHG. A prediction model for PHG was then constructed based on a training dataset that contained data on 492 LC patients. The discrimination, calibration, and clinical utility of the predicting nomogram were assessed using the C-index, calibration plot, and decision curve analysis. Internal validation was conducted using a bootstrapping method, and further external validation using data on the 208 other patients.

**Results:**

LC patients with PHG had a worse prognosis compared with those without PHG. A nomogram was constructed using clinical parameters, such as age, hemoglobin content, platelet count and Child-Pugh class. The C-index was 0.773 (95% CI: 0.730–0.816) in the training cohort, 0.761 after bootstrapping and 0.745 (95% CI: 0.673–0.817) in the validation cohort. The AUC values were 0.767, 0.724, and 0.756 in the training, validation and total cohorts, respectively. Well-fitted calibration curves were observed in the training and validation cohorts. Decision curve analysis demonstrated that the nomogram was clinically useful at a threshold of 15%.

**Conclusion:**

The nomogram constructed to predict the risk of developing PHG was found to be clinically viable. Furthermore, PHG is an independent risk factor for OS of LC, but not for the occurrence of HCC.

## Introduction

Portal hypertensive gastropathy (PHG) is a critical but ignored complication of liver cirrhosis (LC), and is frequently diagnosed using endoscopy through the observation of its characteristic mosaic-like pattern with or without red spots. Histologically, PHG is manifested as dilated capillaries or venules in the mucosa or submucosa of the stomach without erosion, inflammation, or fibrinous thrombi (1). It can be caused by cirrhotic or non-cirrhotic portal hypertension that result in hyperdynamic circulation and gastric congestion, although its exact pathogenesis is unclear (2). The prevalence of PHG has been reported to greatly vary from about 35 to 80% in cirrhotic patients due to the use of different classification criteria and study populations (1).

Esophageal varix (EV) is the most common cause of upper gastrointestinal bleeding in cirrhotic patients with portal hypertension. Addition to EV, PHG is also one of the most common causes of cirrhotic related upper gastrointestinal bleeding which should be took into account. The incidence of PHG-related acute and chronic gastrointestinal bleeding is about 2–12% and 3–60%, respectively (1). Acute bleeding from PHG is less frequent than from varices, but it might be life-threatening as well, and the incidence of bleeding increases as the PHG gets worse. Previous research reported that bleeding-related mortality was up to 12.5% in the PHG because of the diffuse lesion being difficult for treating (3, 4). Moreover, PHG was more prevalent in patients with more fibrosis on liver biopsy (26% in patients with Ishak score of 3 and 51% in patients with Ishak score of 6), which suggested that PHG could be associated with more severe portal hypertension (5, 6).

Although electronic gastroscopy detection (EGD) is the golden standard for the diagnosis of PHG, EGD has not been routinely used for the screening of PHG in patients with cirrhosis due to its invasive procedure, poor tolerance and high cost (1, 7). What's worse, the invasive detection may induce serious esophageal and gastric varices rupture and hemorrhage during the EDG procedure. And the endoscopists usually focus on the varices while underestimate the severity of PHG. However, there is absence of suitable non-invasive technique to assess PHG to date (8–10). Therefore, it is urgent to construct a non-invasive method to screen PHG in patients with cirrhosis.

In this study, we aimed to assess the effect of PHG on LC prognosis, explore the relationship between PHG and other decompensated endpoints of LC such as hepatocellular carcinoma (HCC) incidence, and construct a nomogram model to predict PHG.

## Methods

### Study Patients

This was a retrospective study, and hospitalized patients with liver cirrhosis were admitted to the Department of Gastroenterology, Daping Hospital of Army Medical University in China, between August 2012 and June 2018. Patients with incomplete data or diagnosis of liver cancer at inclusion were excluded. In summary, 700 cirrhotic patients who had undergone gastroscopy during hospitalization were included in the final dataset. Cirrhosis was diagnosed based on a combination of clinical, laboratory and radiological indices, or histological information. The presence and severity of PHG was evaluated using gastroscopy according to the wide-used McCormack standard: “Mild” with features like fine pink speckling (scarlatina-type rash), and mosaic pattern (snakeskin appearance); “Severe” as discrete red spots or diffuse hemorrhagic lesion (11). The patients were followed up regularly in the outpatient department or through a telephone interview to record survival and hepatocarcinogenesis up to July 2019. The primary endpoint was death occurrence, and the secondary endpoint was incidence of HCC.

The study was approved by the Ethics Committee of Daping Hospital, the Third Affiliated Hospital of Army Medical University. All procedures were performed in accordance with the ethical standards of the institutional research committee.

### Clinicopathological Variables

Baseline data including sex, age, the etiology of LC, and laboratory findings were collected. Child-Pugh classification, APRI [aspartate aminotransferase (AST)-to-platelet ratio index], FIB4 and GUCI (Göteborg University Cirrhosis Index) scores for fibrosis and Model for End-stage Liver disease (MELD) score upon hospitalization were also calculated and extracted for each patient (12).

### Statistical Analysis

All statistical analysis were performed using SPSS (version 23.0) for Windows and R (version 3.5.2) software. The normal distribution of continuous variables was assessed using the Shapiro-Wilks test. Non-normally distributed variables were shown as medians and interquartile ranges (IQRs), while categorical ones were expressed as absolute numbers and percentages. The Kruskal-Wallis test was used to compare the continuous variables, while categorical variables were compared using the chi-square test. Survival and HCC development were analyzed using the Kaplan-Meier method and the results were compared using the log-rank test.

The Cox proportional hazard model was used to identify independent prognostic factors for survival, and hazard ratios (HR) with a 95% confidence interval (CI) were calculated. The patients were then randomly divided into two cohorts using a 70:30 ratio (13), and the former was used as the training cohort for nomogram construction. Univariate logistic regression was used to identify factors that were significantly associated with PHG, and the odds ratios (OR) with a 95% CI were calculated. Variables in the univariate logistic regression with a 95% CI that did not cross 1 and *p* < 0.05 were subsequently involved into multivariate logistic regression analysis. The independent risk factors associated with PHG obtained from the multivariate logistic regression were incorporated into the nomogram. A bootstrapping method with 1,000 iterations was used for internal validation (14, 15), and external validation was performed using the remaining 30% of patients. The discriminatory power of the nomogram was expressed in terms of a concordance index (Harrell's C-index) and the area under the curve (AUC) of the receiver operating characteristic (ROC) curve (16, 17), with values closer to 1 (range 0.5–1) indicating higher discrimination ability. Calibration plots were drawn to assess the discrepancy between the real and the nomogram-predicted probabilities (18), which were repeated using the validation cohort. Since AUC alone was not enough for decision-making (19), the clinical utility of the nomogram was evaluated through a decision curve analysis (DCA), which quantified the net benefits at different threshold probabilities (20). All tests were two-tailed, and *P*-values of < 0.05 were considered statistically significant.

## Results

### Patient Characteristics

**Figure 1:**
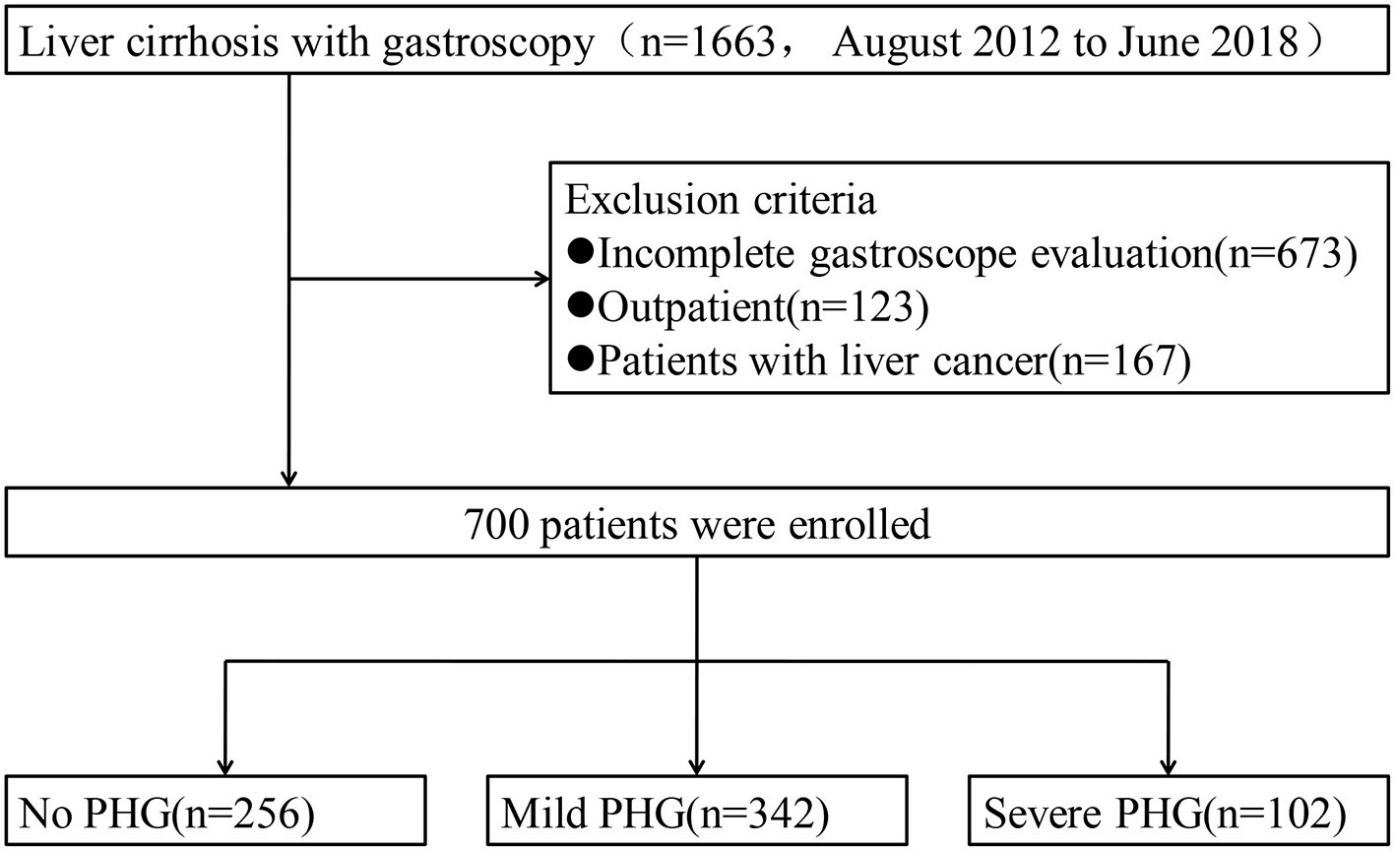
The study flow diagram.

Of the 1,663 patients with liver cirrhosis who had undergone gastroscopy at our hospital from August 2012 to June 2018, 963 were excluded (Figure 1) and the remaining 700 were enrolled in this study. The clinical characteristics of these patients are summarized (Table 1). PHG was observed in 444 (63.4%) patients, of which 342 had mild PHG and 102 had severe PHG, and the respective median age of each group was 53 and 54 years old, respectively. Furthermore, two-thirds (66.1%) of patients were males. Hepatitis virus (HBV/HCV) infection was the most frequent etiological factor, followed by alcohol abuse. Although 38.4% of patients had a history of chronic liver disease spanning more than 5 years, fewer than 100 patients had undergone endoscopic therapies, such as ligation or sclerosis for varices. Child-Pugh classification and MELD scores showed significant differences between the PHG and non-PHG groups, indicating greater severity of LC in the former (*p* < 0.01). In the stratified PHG analysis, multiple laboratory parameters including white blood cell (WBC) and platelet counts were also found to be significantly different, as previously reported (5, 6). APRI, FIB-4 and GUCI are three non-invasive indices of liver fibrosis and cirrhosis that show perfect diagnostic performance (12, 21), and were found to be significantly elevated in the PHG group (Table 1; *p* < 0.001). This is consistent with the results of a previous study that found an association between PHG and advanced fibrosis (5).

**Table 1:** The clinicopathological characteristics of patients enrolled in this study.

**Parameters**	**PHG_No (***n*** = 256)**	**PHG_Mild (***n*** = 342)**	**PHG_Severe (***n*** = 102)**	***P*** **-value**
Age, years	57 (48–67)	53 (46–61)	54 (46–64)	0.018
Sex, *n* (%)				0.44
Male	163 (63.7)	228 (66.7)	72 (70.6)	
Female	93 (36.3)	114 (33.3)	30 (29.4)	
Etiology of cirrhosis, *n* (%)				0.008
Hepatitis virus	152 (59.4)	192 (56.1)	54 (52.9)	
Alcohol	36 (14.1)	44 (12.9)	15 (14.7)	
Hepatitis virus + alcohol	1 (0.4)	25 (7.3)	5 (4.9)	
Others	67 (26.2)	81 (23.7)	28 (27.5)	
Time of CLD, *n* (%)				0.543
<5 y	155 (60.5)	217 (63.5)	59 (57.8)	
≥5 y	101 (39.5)	125 (36.5)	43 (42.2)	
Previous endoscopic treatment*, *n* (%)				0.017
No	230 (89.8)	295 (86.2)	80 (78.4)	
Yes	26 (10.2)	47 (13.8)	22 (21.6)	
WBCs (10^9^/L)	4.29 (3.23–5.79)	3.41 (2.41–5.15)	4.08 (2.71–5.46)	<0.001
Hemoglobin (g/L)	119 (92–139)	96 (76–115)	87 (75–112)	<0.001
Platelets (10^9^/L)	93.5 (61–144)	57 (41–81)	56 (41–75)	<0.001
Total protein (g/L)	66.7 (71.0–73.0)	64.4 (57.1–70.9)	62.9 (57.3–72.2)	0.002
Albumin (g/L)	35.1 (29.5–40.6)	32.2 (28.2–36.6)	33.6 (29.1–37.6)	<0.001
Total bilirubin (mg/dL)	1.38 (0.92–2.08)	1.49 (1.02–2.31)	1.55 (1.08–2.32)	0.257
AST (U/L)	44.4 (28.6–72.8)	44.2 (30.3–65.3)	36.2 (26.2–58.7)	0.061
ALT (U/L)	31.9 (20.3–57.33)	32.8 (21.9–44.8)	27.2 (20.9–42.9)	0.142
ALP (U/L)	103.7 (77.3–143.8)	103.5 (76.9–146.8)	84.2 (71.2–176.4)	0.045
GGT (U/L)	48.6 (24.2–99.9)	44.3 (23.3–96.1)	31.7 (19.6–81.1)	0.064
Urea (mmol/L)	4.8 (4.0–6.3)	5.0 (3.9–7.3)	6.1 (4.5–9.8)	<0.001
Creatinine (mg/dL)	0.76 (0.64–0.90)	0.76 (0.63–0.92)	0.81 (0.71–0.97)	0.017
Uric acid (umol/L)	300 (248–353)	284 (230–348)	315 (261–370)	0.018
INR	1.08 (0.99–1.24)	1.20 (1.06–1.34)	1.21 (1.10–1.42)	<0.001
PT (s)	12.7 (11.4–14.4)	13.8 (12.4–15.7)	13.9 (12.7–16.9)	<0.001
APRI	1.24 (0.59–2.63)	1.98 (1.18–3.34)	1.66 (1.09–3.10)	<0.001
FIB-4	4.95 (2.57–8.48)	7.31 (4.70–11.84)	6.88 (4.86–11.55)	<0.001
GUCI	1.46 (0.61–3.09)	2.23 (1.34–4.24)	2.10 (1.29–4.17)	<0.001
MELD	8.22 (6.52–11.22)	9.42 (7.37–11.87)	9.43 (7.01–13.20)	0.005
Child score	6 (5–8)	7 (6–8)	6.5 (6–8)	<0.001
Child-Pugh, *n* (%)				<0.001
Class A	157 (61.8)	138 (40.3)	51 (50.0)	
Class B	80 (31.2)	152 (44.4)	43 (42.2)	
Class C	19 (7)	52 (15.2)	8 (7.8)	
Splenectomy				0.73
No	248 (96.9)	327 (95.6)	98 (96.1)	
Yes	8 (3.1)	15 (4.4)	4 (3.9)	
Portal vein thrombosis				<0.001
No	205 (80.1)	264 (77.2)	72 (70.6)	
Yes	7 (2.7)	30 (8.8)	15 (14.7)	
Unknown	44	48	15	
**Portal vein diameter (cm)**				
Main	1.40 (1.27–1.56)	1.53 (1.35–1.70)	1.57 (1.37–1.78)	<0.001
Left	0.99 (0.88–1.12)	1.11 (0.99–1.26)	1.10 (0.99–1.31)	<0.001
Right	1.04 (0.91–1.21)	1.11 (0.97–1.29)	1.14 (1.01–1.34)	0.001
Splenic vein diameter (cm)	0.95 (0.79–1.14)	1.16 (1.00–1.41)	1.19 (0.97–1.44)	<0.001
**Splen (cm)**				
Length	13.3 (11.3–15.9)	16.2 (14.1–18.2)	16.2 (14.1–19.1)	<0.001
Width	12.4 (10.6–14.7)	14.4 (12.3–16.3)	14.2 (11.9–17.0)	<0.001
Thickness	5.6 (4.7–6.3)	6.6 (5.8–7.4)	6.7 (6.0–7.7)	<0.001

* *Previous endoscopic treatment means variceal ligation or sclerotherapy ever before*.

### Survival Analysis of Patients With LC

**Figure 2:**
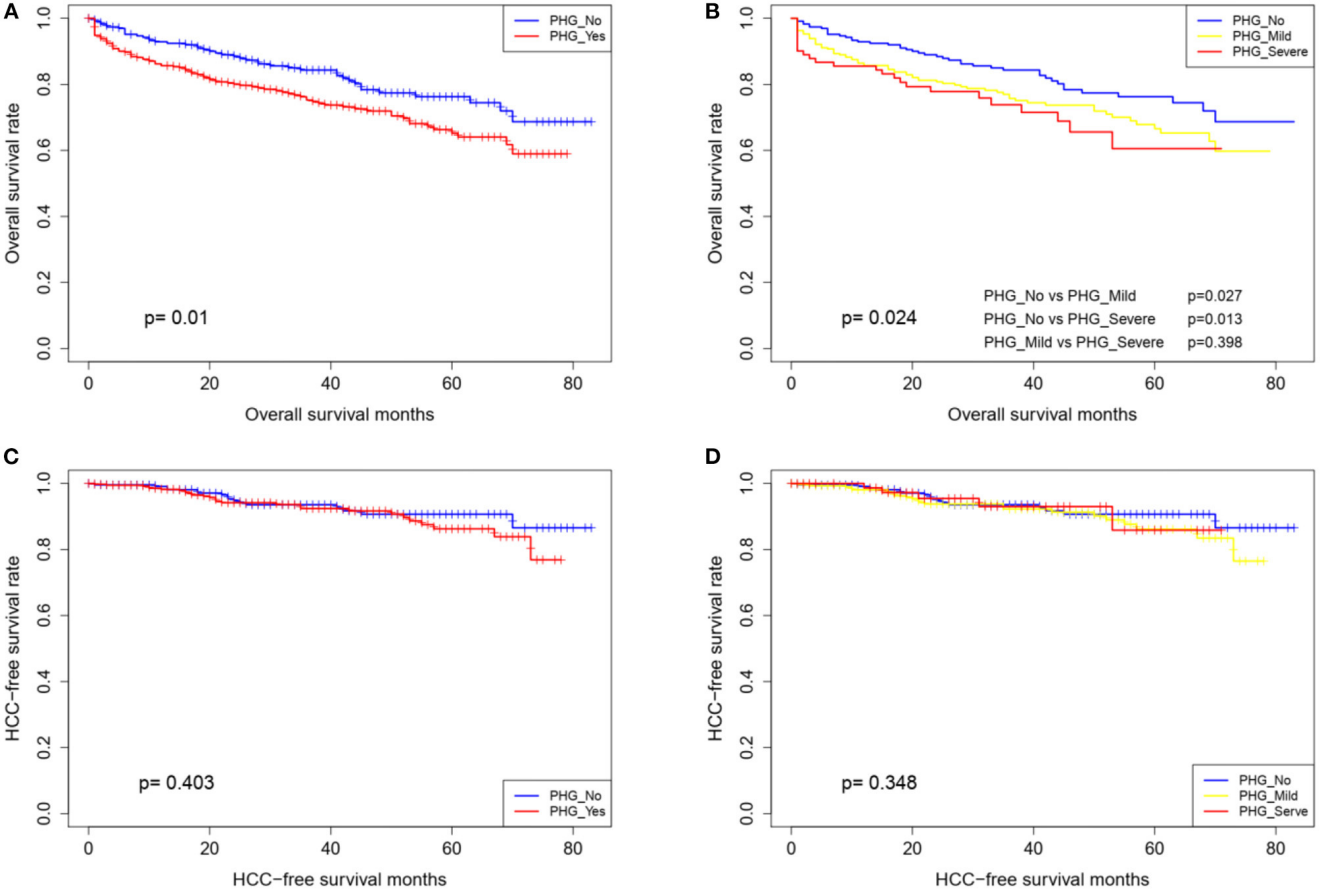
Overall survival and HCC-free survival curves of patients stratified according to the presence **(A,C)** and grade of PHG **(B,D)**.

Eighty patients were lost during the mean follow-up period of 30.7 months. At the end of the follow-up,144 patients had died and 44 had developed HCC. As shown in Table 2, age, PHG, hemoglobin content and MELD scores were found to be associated with poor prognosis, as shown by the univariate and multivariate cox regression analysis. Furthermore, patients with PHG showed worse survival, compared with those without PHG (HR = 1.587, 95% CI: 1.097–2.295, *P* = 0.014), with a mean survival duration of 59 months, compared with 68.2 months for patients without PHG. The survival duration further decreased to 52.2 months in patients with severe PHG. The survival rate of the patients also worsened upon the occurrence and aggravation of PHG (Table 3 and Figure 2), and the 5-year survival rate among those with severe PHG was only 60%. Furthermore, HCC was observed in 16, 23, and 5 patients with no, mild and severe PHG, respectively, during the follow-up period, but was not significantly correlated with the presence or severity of PHG (*p* < 0.05; Figures 2C,D).

**Table 2:** Cox regression analysis demonstrating the association between variables and OS.

**Parameters**	**Univariate analysis**			**Multivariate analysis**		
	**HR**	**95% CI**	***P*** **-value**	**HR**	**95% CI**	***P*** **-value**
Age	1.052	1.037–1.066	<0.001	1.061	1.045–1.078	<0.001
Sex	1.095	0.779–1.540	0.600	NA		
PHG (yes vs. no)	1.587	1.113–2.264	0.011	1.587	1.097–2.295	0.014
Etiology of cirrhosis	1.256	1.074–1.470	0.004	1.066	0.899–1.264	0.465
Time of CLD	0.803	0.570–1.132	0.211	NA		
Previous endoscopic treatment	1.188	0.761–1.856	0.448	NA		
WBCs (10^9^/L)	1.072	1.020–1.127	0.006	1.050	0.997–1.106	0.064
Hemoglobin	0.988	0.982–0.993	<0.001	0.987	0.980–0.994	<0.001
Platelets (10^9^/L)	0.999	0.997–1.002	0.621	NA		
AST	1.000	0.998–1.001	0.715	NA		
ALT	0.998	0.995–1.001	0.162	NA		
APRI	1.035	1.013–1.058	0.002	1.073	0.992–1.161	0.078
FIB-4	1.013	1.009–1.018	<0.001	1.001	0.989–1.013	0.848
GUCI	1.027	1.014–1.040	<0.001	0.960	0.908–1.015	0.155
MELD	1.078	1.045–1.112	<0.001	1.092	1.040–1.147	<0.001
Child-Pugh class	1.879	1.491–2.369	<0.001	1.314	0.992–1.740	0.057
Splenectomy	0.366	0.091–1.476	0.158	NA		

**Table 3:** Survival rate based on PHG presence and grade.

**PHG grade**	**Follow-up months**		**Mean survival**		**Overall survival rate**					
	**Mean ±SD**	**Median**	**Months**	**95% CI**	**1-year**	**95% CI**	**3-year**	**95% CI**	**5-year**	**95% CI**
PHG_No	35.2 ± 23.6	35	68.2	64.4–72.1	0.929	0.896–0.963	0.843	0.794–0.895	0.763	0.697–0.834
PHG_Yes	28.1 ± 23.1	24.5	59.0	55.8–62.3	0.857	0.822–0.893	0.752	0.706–0.802	0.652	0.589–0.721
PHG_Mild	28.8 ± 23.9	25	59.8	56.1–63.4	0.857	0.817–0.898	0.757	0.705–0.814	0.666	0.597–0.743
PHG_Severe	25.9 ± 20.6	23	52.2	46.0–58.3	0.855	0.786–0.931	0.738	0.645–0.845	0.605	0.472–0.776

### Comparison Between the Training and Validation Cohort

Of the 700 patients, 492 were assigned to the training cohort to be used to construct the nomogram, and 208 were assigned to the validation cohort to be used for external model validation. Based on their clinical significance, the continuous variables were converted into categorical variables. Baseline data between the training and validation cohorts showed no significant differences (Table 4).

**Table 4:** Baseline characteristics of the training and validation cohorts.

**Parameters**		**Cohort, no. (%)**		***P*** **-value**
		**Training (***n*** = 492)**	**Validation (***n*** = 208)**
Age	<50 y	175 (35.6)	61 (29.3)	0.189
	<60 y	143 (29.1)	60 (28.8)	
	60+	174 (35.4)	87 (41.8)	
Sex	Male	323 (65.7)	140 (67.3)	0.672
	Female	169 (34.3)	68 (32.7)	
Etiology of cirrhosis	Alcohol	66 (13.4)	29 (13.9)	0.844
	Hepatitis virus	284 (57.7)	114 (54.8)	
	Hepatitis virus+Alcohol	20 (4.1)	11 (5.3)	
	Others	122 (24.8)	54 (26.0)	
Time of CLD	<5 y	310 (63.0)	121 (58.2)	0.229
	≥5 y	182 (37.0)	87 (41.8)	
Previous endoscopic treatment	No	420 (85.4)	185 (88.9)	0.207
	Yes	72 (14.6)	23 (11.1)	
WBCs (10^9^/L)	<4	262 (53.3)	108 (51.9)	0.748
	4+	230 (46.7)	100 (48.1)	
Hemoglobin (g/L)	<90	187 (38.0)	78 (37.5)	0.692
	<120	166 (33.7)	65 (31.3)	
	120+	139 (28.3)	65 (31.3)	
Platelets (10^9^/L)	<100	355 (72.2)	159 (76.4)	0.241
	100+	137 (27.8)	49 (23.6)	
AST (U/L)	<40	218 (44.3)	94 (45.2)	0.830
	40+	274 (55.7)	114 (54.8)	
ALT (U/L)	<40	322 (65.4)	134 (64.4)	0.795
	40+	170 (34.6)	74 (35.6)	
Child-Pugh class	A	245 (49.8)	101 (48.6)	0.956
	B	192 (39.0)	83 (39.9)	
	C	55 (11.2)	24 (11.5)	
Splenectomy	No	471 (95.7)	202 (97.1)	0.385
	Yes	21 (4.3)	6 (2.9)	
PHG	No	181 (36.8)	75 (36.1)	0.854
	Yes	311 (63.2)	133 (63.9)	

### Nomogram Development

**Figure 3:**
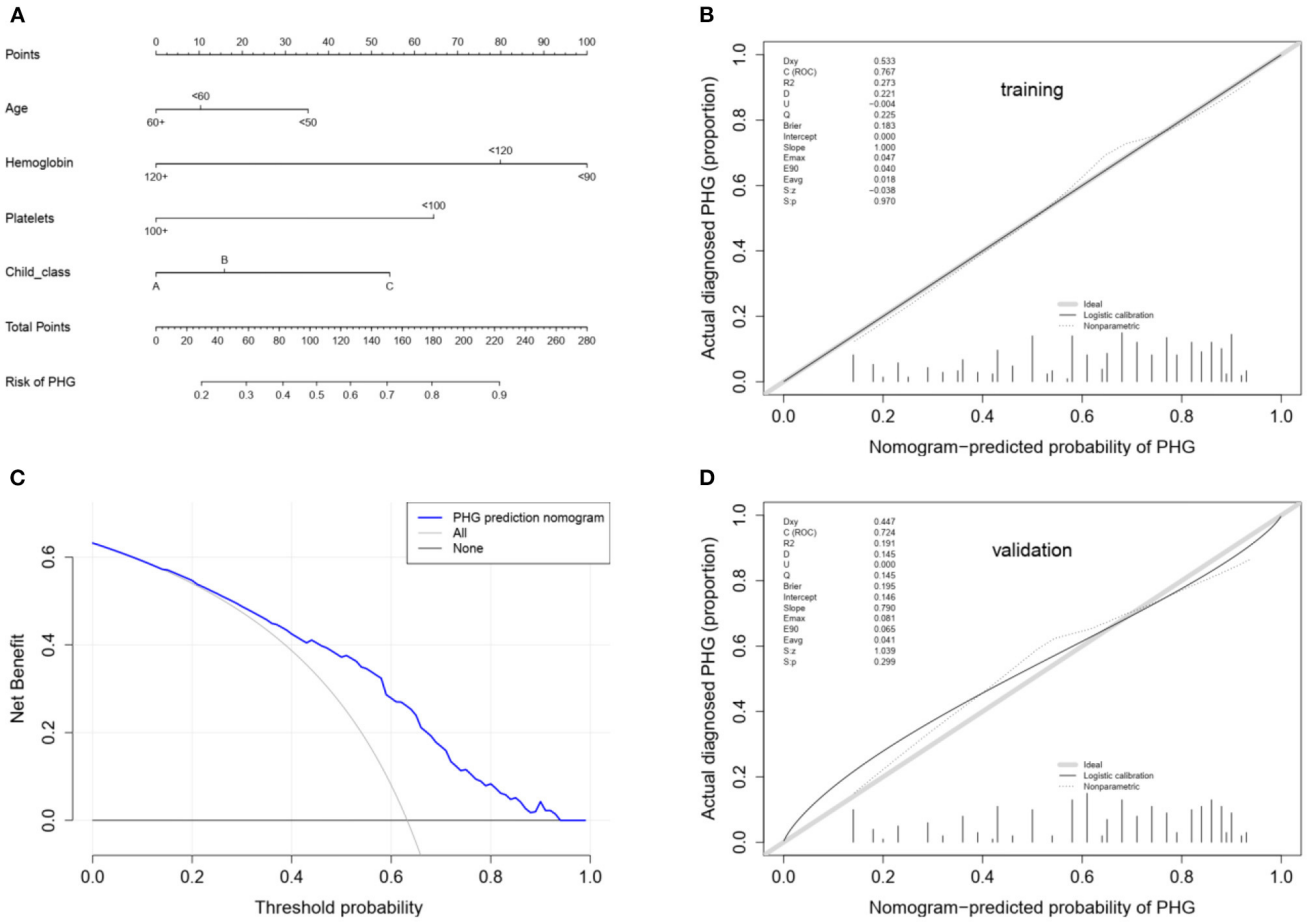
**(A)** Nomogram for the prediction of PHG probability in LC patients. In the nomogram model, the patient's score for each value is located on each variable axis, and a vertical line is drawn upward to determine the number of points received for each variable value. The sum of all variables located on the total points axis and a line drawn downwards to the risk axes are used to determine the probability of the the presence of PHG. **(B)** Calibration using the training cohort. The x-axis represents the predicted probability and the y-axis represents the observed fraction obtained through gastroscopy. Points below the ideal line represent over-prediction, and those above represent under-prediction. The calibration line indicates nomogram performance. **(C)** Decision curve analysis. The y-axis represents the net benefit. The blue line represents PHG risk, the thin solid line represents the assumption that all patients have PHG, and the thick solid line represents the assumption that none of the patients have PHG. **(D)** Calibration using the validation cohort.

The univariate logistic regression analysis of the training group showed that age, WBC count, hemoglobin content, platelet count and Child-Pugh class (*p* < 0.05) were the significant risk factors for PHG. The non-conditional binary multivariate logistic regression analysis of the above revealed that all factors, excluding WBC count were independent risk factors for PHG (Table 5), and were included in the individualized nomogram prediction model (Figure 3A). The predicted risk corresponding to the total score was calculated as the sum of each indicator. The nomogram showed that hemoglobin content was the largest contributor to the risk score, followed by platelet count and Child-Pugh class.

**Table 5:** Logistic regression analysis on the training cohort.

**Parameters**	**Univariate analysis**			**Multivariate analysis**		
	**OR**	**95% CI**	***P*** **-value**	**OR**	**95% CI**	***P*** **-value**
Age	0.767	0.616–0.956	0.018	0.745	0.582–0.955	0.020
Sex	0.830	0.566–1.219	0.342	NA		
Etiology of cirrhosis	0.973	0.811–1.168	0.768	NA		
Time of CLD	0.998	0.683–1.459	0.993	NA		
Previous endoscopic treatment	1.616	0.931–2.807	0.088	NA		
WBCs (10^9^/L)	0.647	0.448–0.935	0.021	NA		
Hemoglobin (g/L)	0.376	0.292–0.483	0.000	0.402	0.308–0.524	0.000
Platelets (10^9^/L)	0.271	0.180–0.409	0.000	0.296	0.189–0.463	0.000
AST	0.861	0.595–1.247	0.429	NA		
ALT	0.697	0.475–1.020	0.063	NA		
Child-Pugh class	1.882	1.403–2.525	0.000	1.574	1.148–2.160	0.005
Splenectomy	1.478	0.563–3.880	0.427	NA		

### Nomogram Performance

**Figure 4:**
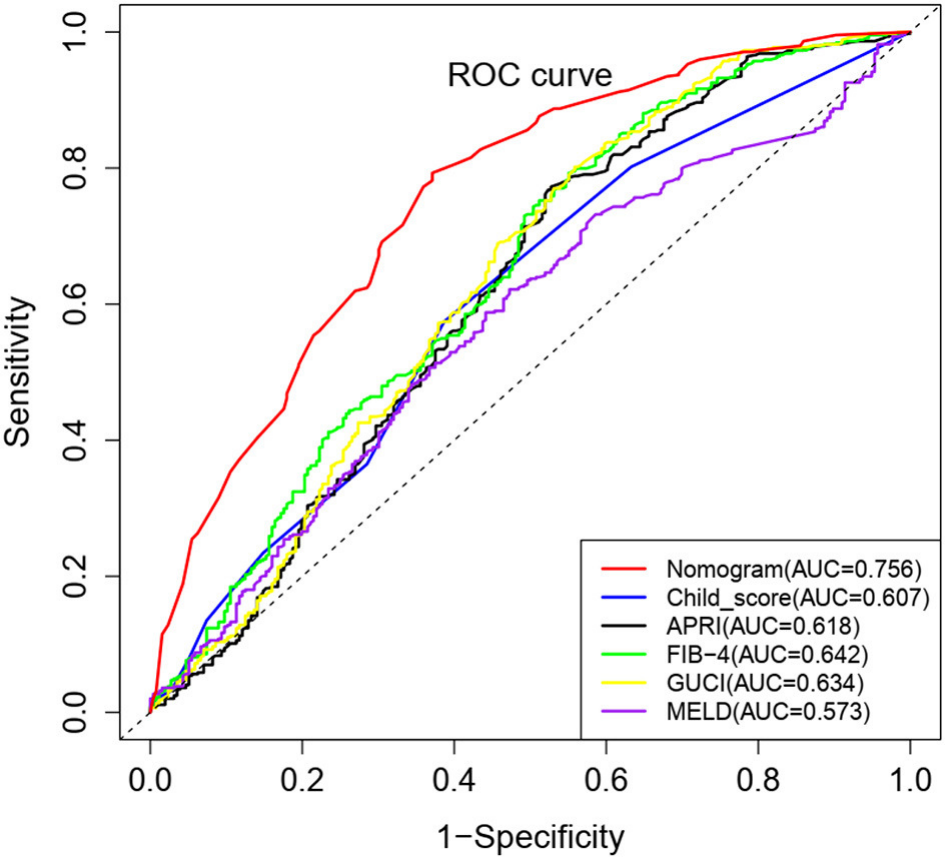
Comparison of ROC curves for different non-invasive parameters using the complete cohort. The nomogram model has the highest diagnostic accuracy with an AUC value 0.756.

The nomogram model was further evaluated regarding its discrimination, calibration and clinical utility abilities. The C-index was 0.773 (95% CI: 0.730–0.816), 0.761 and 0.745 (95% CI: 0.673–0.817) in the training cohort, after internal validation through bootstrapping with 1,000 iterations and in the validation cohort, respectively. Statistically similar C-indices between the training and validation cohorts (*P* = 0.745) indicated that the model was reproducible. The sensitivity, specificity, positive predictive value (PPV), negative predictive value (NPV) and accuracy for the nomogram in the training cohort were 88.7, 48.6, 74.8, 71.5, and 74.0%, respectively. Furthermore, the AUCs for the training and validation cohorts were 0.767 and 0.724, respectively, and that of the entire cohort was 0.756. The ROC curve was also used to compare the AUC values of other factors including APRI, FIB-4, GUCI, Child-Pugh class, and MELD score. The nomogram model gave the best satisfactory distinction (Figure 4). However, AUC values of < 0.65 obtained for each indicated that none of these factors can be used to effectively diagnose PHG. The calibration plots for the training and validation groups are shown in Figures 3B,D, and both were similar to the ideal curve. Furthermore, the *P*-values of 0.970 and 0.299 obtained in the calibration test showed that the predictive ability of the nomogram was also close to the actual probability. The decision curves for the training cohort are shown in Figure 3C, and indicated that the model can be used to accurately predict PHG in cirrhotic patients with a threshold probability ranging from 15 to 93%.

## Discussion

Although EGD is the golden standard for the diagnosis of PHG, endoscopic screening is limited owing to its invasive operation, high cost and the need for anesthetic. Thus, non-invasive methods for PHG diagnosis are needed. However, visualized non-invasive model for PHG predicting has not been reported before. In this study, we firstly developed and validated a visualized, non-invasive, clinical parameter-based nomogram for the prediction of PHG in cirrhosis.

Nomograms are widely used in oncology and medicine as prognostic/diagnostic model, and their user-friendly visual interfaces and accurate predictive performance are greatly beneficial for clinical decision making (22, 23). To the best of our knowledge, this study was the first to apply a nomogram for the prediction of PHG. We developed and validated a relatively accurate predictive nomogram to be used as a non-invasive diagnostic tool for PHG in LC patients using four easily measurable variables. The prognostic implications of PHG on overall and HCC-free survival were firstly assessed, which indicated significantly worse survival in patients with PHG and corresponded with the stratified analysis of PHG severity. This finding is consistent with that of two previous studies (24, 25). However, the prognosis of the mild and severe PHG groups were similar (*p* = 0.398), which was likely due to the relatively small sample size of the latter. In agreement with a previous study (25), PHG showed no predictive value for HCC incidence, which may be attributed to the involvement of multiple oncogenic factors (26).

The MELD score, Child-Pugh class and indicators of portal vein pressure such as portal/splenic vein diameter, thrombosis, and spleen size, were relatively worse in patients with PHG, which is consistent with previous reports (5, 24, 27). Therefore, PHG was found to be a potential indicator of advanced hepatic disease and poor prognosis in LC patients. In this study, 63% of the cirrhotic patients presented with PHG, which is similar to the results of previous reports (27–29). Age, hemoglobin content, platelet count and Child-Pugh class were identified as independent risk factors for PHG. Lower hemoglobin content and platelet count, younger age and greater severity of liver disease were associated with an increased risk of PHG, which is consistent with the results of previous studies (1, 5, 24). Subsequently, we developed an effective risk prediction tool to identify patients at risk of PHG, which showed high sensitivity, PPV, NPV, and accuracy. The non-invasive clinical predictors PSR and RLAR were similarly evaluated in a recent study, and showed an accuracy of 74.7 and 35.1%, and relatively low levels of specificity and NPVs (30). In contrast, our nomogram showed satisfactory discrimination, calibration and clinical utility value. As far as we know, our study is also the first report to assess the diagnostic accuracy of APRI, FIB-4, and GUCI to detect PHG. Interestingly, all the AUC values of APRI, FIB-4, GUCI, Child-Pugh class and MELD score were not satisfactory. This may be due to patient heterogeneity caused by various cirrhosis etiologies.

It should be pointed out that our study has several limitations. First of all, as a retrospective study, the associated certainty of patient information is limited. Second, this study was conducted on the inpatients at a single center, which may not be representative of all cirrhotic patients. Furthermore, factors that may potentially affect PHG, such as blood ammonia level, were not included in the risk factor analysis (28). Therefore, the generalizability of the study findings needs to be further validated through a prospective multicenter study conducted on a larger cohort of patients.

In conclusion, we developed and validated a visualized, clinical parameter-based nomogram which is a reliable and non-invasive method to predict PHG in cirrhotic patients. Besides, we also explored the relationship between PHG and other decompensated endpoints of LC such as HCC incidence. PHG is an independent risk factor for OS of LC, but not for the occurrence of HCC. PHG and HCC might be independent decompensated endpoint events of LC which suggests PHG cannot be used as an indicator for HCC surveillance.

## Data Availability Statement

The original contributions presented in the study are included in the article/supplementary material, further inquiries can be directed to the corresponding author.

## Ethics Statement

The studies involving human participants were reviewed and approved by the Ethics Committee of Daping Hospital. Written informed consent for participation was not required for this study in accordance with the national legislation and the institutional requirements.

## Author Contributions

WW and ZM prepared the manuscript. LW and DC designed and drafted the manuscript. WW, ZM, GZ, TW, SL, YG, and XY were involved in data curation and investigation. WW, ZM, GZ, LW, and DC contributed to the interpretation of the results. TW, SL, YG, and XY were responsible for data analyzing and visualization. All authors contributed to the article and approved the submitted version.

## Funding

This work was supported by National Natural Science Foundation of China (NCFS:81802459), Chongqing Natural Science Foundation (cstc2018jcyjAX0603), and Sponsored by Science and Technology Innovation Enhancement Project of Army Medical University (2019XLC3045).

## Conflict of Interest

The authors declare that the research was conducted in the absence of any commercial or financial relationships that could be construed as a potential conflict of interest.

## Publisher's Note

All claims expressed in this article are solely those of the authors and do not necessarily represent those of their affiliated organizations, or those of the publisher, the editors and the reviewers. Any product that may be evaluated in this article, or claim that may be made by its manufacturer, is not guaranteed or endorsed by the publisher.
